# Cellular engineering of *Artemisia annua* and *Artemisia dubia* with the *rol ABC* genes for enhanced production of potent anti-malarial drug artemisinin

**DOI:** 10.1186/s12936-016-1312-8

**Published:** 2016-05-04

**Authors:** Bushra Hafeez Kiani, John Suberu, Bushra Mirza

**Affiliations:** Department of Biochemistry, Quaid-i-Azam University, Islamabad, Pakistan; Department of Life Sciences, University of Warwick, Coventry, CV4 7AL UK; Department of Chemical Engineering and Biotechnology, University of Cambridge, Cambridge, UK

**Keywords:** *Artemisia annua*, *Artemisia dubia*, *rolABC* genes, Artemisinin, *Agrobacterium tumefacienes*, *Agrobacterium rhizogenes*

## Abstract

**Background:**

Malaria is causing more than half of a million deaths and 214 million clinical cases annually. Despite tremendous efforts for the control of malaria, the global morbidity and mortality have not been significantly changed in the last 50 years. Artemisinin, extracted from the medicinal plant *Artemisia* sp. is an effective anti-malarial drug. In 2015, elucidation of the effectiveness of artemisinin as a potent anti-malarial drug was acknowledged with a Nobel prize. Owing to the tight market and low yield of artemisinin, an economical way to increase its production is to increase its content in *Artemisia* sp. through different biotechnological approaches including genetic transformation.

**Methods:**

*Artemisia annua* and *Artemisia dubia* were transformed with *rol ABC* genes through *Agrobacterium tumefacienes* and *Agrobacterium rhizogenes* methods. The artemisinin content was analysed and compared between transformed and untransformed plants with the help of LC–MS/MS. Expression of key genes [Cytochrome P450 (*CYP71AV1*), aldehyde dehydrogenase 1 (*ALDH1*), amorpha-4, 11 diene synthase (*ADS*)] in the biosynthetic pathway of artemisinin and gene for trichome development and sesquiterpenoid biosynthetic (*TFAR1*) were measured using Quantitative real time PCR (qRT-PCR). Trichome density was analysed using confocal microscope.

**Results:**

Artemisinin content was significantly increased in transformed material of both *Artemisia* species when compared to un-transformed plants. The artemisinin content within leaves of transformed lines was increased by a factor of nine, indicating that the plant is capable of synthesizing much higher amounts than has been achieved so far through traditional breeding. Expression of all artemisinin biosynthesis genes was significantly increased, although variation between the genes was observed. *CYP71AV1* and *ALDH1* expression levels were higher than that of *ADS*. Levels of the *TFAR1* expression were also increased in all transgenic lines. Trichome density was also significantly increased in the leaves of transformed plants, but no trichomes were found in control roots or transformed roots. The detection of significantly raised levels of expression of the genes involved in artemisinin biosynthesis in transformed roots correlated with the production of significant amounts of artemisinin in these tissues. This suggests that synthesis is occurring in tissues other than the trichomes, which contradicts previous theories.

**Conclusion:**

Transformation of *Artemisia* sp. with *rol ABC* genes can lead to the increased production of artemisinin, which will help to meet the increasing demand of artemisinin because of its diverse pharmacological and anti-malarial importance.

## Background

Artemisinin is a secondary metabolite that has been found to have strong anti-malarial activity with little or no side effects [1]. Artemisinin is a sesquiterpene lactone which has been reported to be produced within the glandular trichomes of *Artemisia annua*, a member of the Asteraceae family. This plant is endemic to China and has been used there for over 2000 years to treat fever [2, 3]. Artemisinin has also been found in aerial parts of *Artemisia apiacea, Artemisia lancea* and *Artemisia cina* [3–7] have also reported the presence of artemisinin in *Artemisia dubia*, *Artemisia absinthium* and *Artemisia indica.*

At present, artemisinin and its derivatives are used in combination therapies for the treatment of malaria [8], and for the treatment of a number of cancers and viral diseases [9, 10]. However, these therapies are not available to millions of the world’s poorest people because of the low yield of artemisinin in naturally growing *Artemisia* plants (0.1 to 0.5 % of dry weight) [11]. Mannan et al. [12] reported the presence of artemisinin in 17 naturally growing species of *Artemisia* and found artemisinin content in the range of 0.03 to 0.44 %. Enhanced production of artemisinin is, therefore, highly desirable. Genetic improvement of natural varieties has been attempted, but the maximum yield of artemisinin achieved so far is 2 % [13]. Even though there are some recent reports about cost effective chemical synthesis of artemisinin [14] or through microbial system [15], these methods are not yet practical for large-scale production of artemisinin. Due to complexity in production [8, 16], biosynthesis of artemisinin via the plant is still the preferred choice. If yields could be improved significantly, the market price could be stabilized and the drug made available at lower cost. One approach is to optimize production though modification of the metabolic pathways involved, this could be through selection for beneficial alleles or through genetic transformation.

**Fig. 1:**
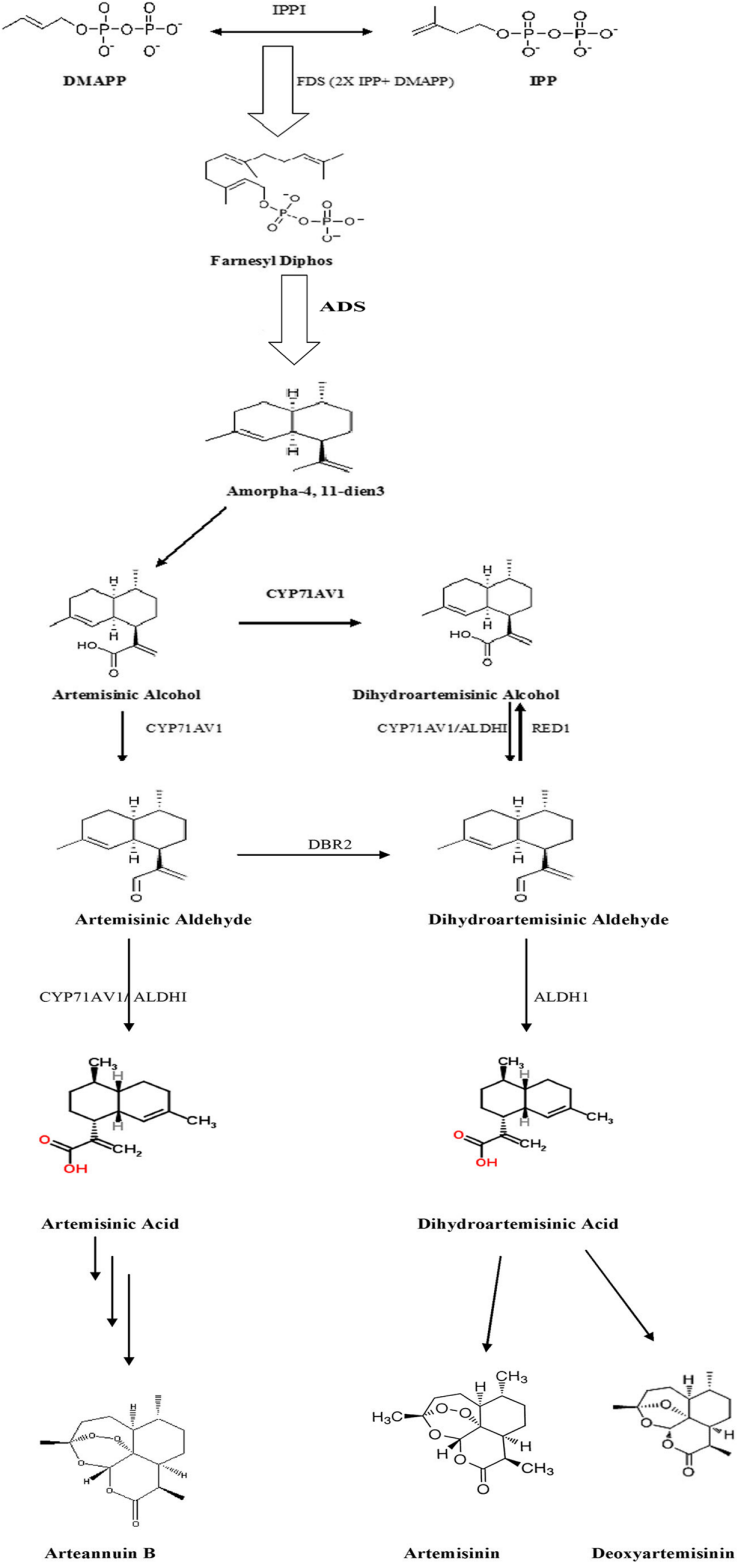
Biosynthetic pathway of artemisinin. Figure showing pathway and key genes and enzymes involved in artemisinin biosynthesis [17]. ADS-Amorpha-4, 11-diene synthase, ALDH- Aldehyde dehydrogenase, CYP-CYP71AV1 (p450 enzymes), Dbr2 -Artemisinic aldehydeD11 (13) double bond reductase, DMAPP- Dimethyl allyl diphosphate, IPP- Isopentenyl diphosphate

Several groups have shown that transformation with different genes can increase artemisinin production through modification of its biosynthetic pathway. Overexpression of FPS in *Artemisia annua* increases the accumulation of artemisinin through conversion of IPP and DMADP into FDP (Fig. 1) [17]. Biswajit et al. [18] reported an increased amount of artemisinin in *Artemisia annua* transformed with *Agrobacterium tumefaciens* wild-type nopaline strains. Sa et al. [19] transferred the *Ipt* gene into *Artemisia annua* and observed increased amount of artemisinin in transformed plants. Over the past decade, increased production of secondary metabolites has been shown in plant cells transformed with the *rol A*, *B* and *C* genes. The *rol A* protein has emerged as a stimulator of growth and secondary metabolism [20, 21]. The *rol B* protein on the other hand, has been shown to have tyrosine phosphatase activity and, therefore, may play a role in the auxin signal transduction pathway [22]. Estruch et al. [23] demonstrated that *rol C* can be involved in the release of active cytokinins from their inactive glucosides due to its cytokinin glucosidase activity. *rol C* has also been shown in transformed plants and plant cell cultures to be capable of stimulating the production of tropane alkaloids [24], pyridine alkaloids [25], indole alkaloids [26], ginsenosides [27] and anthraquinones [28, 29].

The first step in artemisinin biosynthesis (Fig. 1) is the generation of amorpha-4, 11-diene by the cyclization of farnesyl diphosphate (FDP), catalyzed by amorpha-4, 11-diene synthase (ADS) [30]. Subsequent oxidation at the C^12^ position, mediated by the cytochrome P450 enzyme CYP71AV1, leads to artemisinic alcohol [31, 32]. While arteannuin B has been suggested as a late precursor in artemisinin biosynthesis [33, 34], evidence now favours a route from artemisinic alcohol via dihydroartemisinic acid [2, 35, 36].

Cloning and characterization of double bond reductase 2 (DBR2) supported this route, which reduces the D11 (13) double bond of artemisinic aldehyde, but not of arteannuin B [34], and the cloning of aldehyde dehydrogenase 1 (ALDH1), which catalyses the oxidation of artemisinic and dihydroartemisinic aldehyde [32]. According to Brown et al. [37–39], the conversion of dihydroartemisinic acid to artemisinin, and of artemisinic acid to arteannuin B occur via enzyme-independent reactions.

A number of publications have reported large differences in artemisinin content depending on variety, season, different plant parts, co cultivation conditions and plant developmental stage [30, 40–45]. Artemisinin has been detected in leaves; small green stems, buds, flowers and seeds [40, 45–47]. Reports on the distribution of artemisinin throughout the plant are however inconsistent. In some studies artemisinin has been reported to be higher at the top of the plant while others suggest it is equally distributed [48]. Artemisinin has also been shown to be produced by differentiated shoot cultures [46, 49, 50], but in shoots without roots, only trace levels are found, suggesting a regulatory effect [49–53]. Most workers [49, 50, 54, 55] have been unable to detect artemisinin in roots, although Nair et al. [56] and Tawfik et al. [54] reported trace amounts.

The current understanding is that artemisinin is produced in ten-celled glandular trichomes located on leaves, floral buds, and flowers [40, 57, 58] and sequestered in the epicuticular sac at the apex of the trichome [58]. For instance, artemisinin concentrations are higher in leaves that are formed later in development than in leaves formed early in the plant’s development; this difference has been attributed to a higher trichome density and a higher capacity per trichome in the upper leaves [59]. Artemisinin was thought not to be synthesized in roots [40] or pollen. ELISA analysis has shown that green roots can accumulate artemisinin (0.001 % dry weight), which was confirmed by GC–MS analysis [60]. Nguyen et al. [16] have also confirmed the presence of artemisinin in roots. It has also been shown that hairy roots produced by infection with *Agrobacterium rhizogenes* can produce artemisinin [60, 61]. Weathers et al. [62] reported levels of artemisinin (0.4 %) comparable to that found in the leaves within hairy root cultures of *Artemisia annua* transformed with *Agrobacterium rhizogenes.* This would suggest that the plant may be capable of producing artemisinin in the absence of trichomes.

Although the artemisinin concentration appears to be greatest in *Artemisia annua*, there are reports describing its presence in *Artemisia absinthium, Artemisia dubia* and *Artemisia indica* [5–7]. Chemical synthesis of artemisinin is difficult and, therefore, other *Artemisia* species or alternative sources, such as plant callus [63], shoot cultures [49] and hairy root cultures [64] offer attractive alternatives. These could be grown throughout the year, distancing production from time of harvest. Mannan et al. [7] found that *Agrobacterium rhizogenes* transformation increased the artemisinin content within hairy roots of *Artemisia dubia* to 0.6 % and to 0.23 % in *Artemisia indica*. *Artemisia annua* is restricted to certain global regions due to its growth requirements. *Artemisia dubia* is capable of growth in more varied conditions and could offer an alternative for production, ensuring security of production which would help ensure a constant price.

Numerous investigations have shown that the *Agrobacterium rol* genes can induce high levels of secondary metabolites in hairy root cultures of most transformed plant species. Data have been published about the effect of individual *rol A, B* and *C* genes on enhanced production of anti-malarial artemisinin [65–68].

The objective of the current study was the assessment of artemisinin production in transformed plants and hairy roots of *Artemisia dubia* and *Artemisia annua* transformed with *rol ABC* genes using *Agrobacterium tumefaciens* and *Agrobacterium rhizogenes* and their comparison with non-transformed plants. Trichome densities and the gene transcripts involved in the synthesis pathway were monitored to ascertain how the *rol* genes act to influence the production of anti-malarial artemisinin.

## Methods

### Seed sterilization and germination

*Artemisia annua* and *Artemisia dubia* seeds from a Northern Pakistan collection were disinfected with 70 % (v/v) ethyl alcohol for 30 s; surface sterilized with 0.1 % (w/v) Mercuric chloride (HgCl_2_) solution for 2 min, washed 3–4 min with sterile distilled water and transferred to half MS medium [69] containing 3 % (w/v) sucrose solidified with 0.8 % (w/v) agar. The seedlings were grown in growth room at 26 °C ± 2 with 16/8 h light dark cycle and light intensity maintained at 1000 lux.

### Bacterial strains

*Agrobacterium tumefaciens* strain LBA4404 containing pRT99 was grown overnight in 50 ml of liquid malt yeast broth (MYB; 5 g/l yeast extract, 0.5 g/l casein hydrolysate, 8 g/l mannitol, 2 g/l ammonium sulfate, 5 g/l sodium chloride, pH 6.6). *Agrobacterium rhizogenes* strains LBA 9402 and 8196 containing Ri plasmid were grown overnight in 50 ml of liquid yeast mannitol broth (YMB; 0.5 g/l Di-potassium hydrogen phosphate, 2 g/l magnesium sulphate, 0.4 g/l yeast extract, 10 g/l mannitol, 0.1 g/l sodium chloride, pH 7.0). Prior to infection, the bacterial strains were grown overnight in liquid medium at 28 °C on a rotary shaker at 100 rpm in the dark.

### *Agrobacterium tumefaciens* mediated transformation

Approximately 0.5–1 cm long stem and leaf explants were excised from the in vitro grown seedlings and were cultured on plain MS medium [69]. After 2–3 days of preculturing, these explants were dipped in *Agrobacterium tumefaciens* culture containing 50 mg/L kanamycin with different time durations; blotted on sterilize filter paper and cocultivated on MS medium containing 200 µM acetosyringone and 0.8 % agar.

After 3 days of cocultivation, the infected explants were washed with cefotaxime solution (500 µg/L), dried and transferred to selection MS medium containing 0.1 mg/L BAP (benzyl aminopurine), 20 mg/L kanamycin and 500 mg/L cefotaxime. These explants were maintained in growth room at 26 °C ± 2 with 16 h of photoperiod, illumination of 45 µE m^−2^s^−1^ and 60 % relative humidity. The explants were then transferred to fresh selection medium weekly during the 1 st month. Afterwards, subculturing was carried out every 2 weeks. After 8 weeks, the concentration of cefotaxime was reduced to 50 mg/L.

For rooting, the developed shoots cut off segments of *Artemisia dubia* were cultured on half MS medium containing 0.025 mg/L naphthalene acetic acid (NAA) solidified with 0.1 % gelrite and developed shoots cut off segments of *Artemisia annua* were cultured on MS medium containing 0.1 mg/L naphthalene acetic acid (NAA) solidified with 0.1 % gelrite.

### *Agrobacterium rhizogenes* mediated transformation

#### Hairy roots induction

The four-week old plants of *Artemisia dubia* and *Artemisia annua* were infected with *Agrobacterium rhizogenes* strain LBA9402 and LBA8196. The stem portions were infected with 24 h old single colony of *Agrobacterium rhizogenes* with the help of scalpel. After production of hairy roots, 3–4 cm root tips were cut and placed on solid B5 medium for further proliferation.

#### Molecular analysis

For molecular analysis, genomic DNA from both *Artemisia* species was isolated by using CTAB method of [70] from transformed and untransformed plants, and plasmid DNA was also isolated by using alkaline lysis method from both *Agrobacterium tumefaciens* and *Agrobacterium rhizogenes.* PCR analysis was performed using a programmed DNA thermal cycler (Biometra, USA). The *rol A* gene forward 5′-AGAATGGAATTAGCCG GACTA-3′ and reverse primer 5′-GTATTAATCCCGTAGGTTTGTT-3′, the *rol B* forward 5′-GCTCTTGCAGTGCTAGATTT-3′ and reverse primer 5′-GAAGGTGC AAGCTACCT CTC-3′, the *rol C* gene forward 5′-GAAGACGACCTGTGTTCTC-3′ and reverse primer 5′-CGTTCAAACGTTAGCCGATT-3′ were used for PCR analysis. The PCR reaction was carried out in 25 µl final reaction volume containing 50 ng DNA template with the following thermal cycling conditions: 35 cycles of 5 min at 94 °C, 1 min at 53–55 °C and 1 min at 72 °C. Agarose gel (1.5 % w/v) electrophoresis was carried out to analyse 10 µl aliquot of PCR product.

Southern blot analysis of PCR-positive plants was performed by DIG High Prime DNA Labeling and Detection Starter Kit II (Roche Cat. No. 11585614910) according to the manufacturer’s instructions. For Southern blotting, 20 µg of genomic DNA was digested with EcoRI and electrophoresed on 0.8 % agarose gel. The DNA was denatured by alkaline solution and transferred to positively charged nylon membranes according to the standard procedure of PCR [96]. Products of *rol A* gene from plasmid was used as the probe. The probe was labeled using digoxigenin (DIG)-11-dUTP with DIG High Prime DNA labelling reagents (Roche, Mannheim, Germany). Hybridization was carried out at 42–45 °C followed by immunological detection on X-ray film using CSPD substrate according to the manufacturer’s instructions.

### Analysis of artemisinin content

#### Chemicals

Reference standard of artemisinin (98 %), n-Hexane, ethyl acetate, LC–MS grade 0.1 % formic acid in water and acetonitrile was obtained from Sigma-Aldrich (Dorset, UK). Purified water (~18 MΩ/cm) was dispensed from a Milli-Q system (Millipore, UK).

#### Plant samples

Shoots, roots and hairy roots of transformed and untransformed plants of *Artemisia annua* (A1, A2, A3, AC, AR1, AR2, AR3, ARC, AH1, AH2) and *Artemisia dubia* (D1, D2, D3, DC, DR1, DR2, DR3, DRC, DH1, DH2) were used for the analysis of artemisinin with the help of LC–MS/MS.

#### Preparation of analytical standards

A standard stock solution of 1 mg mL^−1^ of artemisinin in acetonitrile was prepared. The analytical standard was prepared as dilution from the stock in the mobile phase in the concentration range between 0.15–10 µg mL^−1^. Beta-artemether was used as internal standard (IS) at 5 µg mL^−1^.

#### Sample extraction and preparation

Extraction of plant samples (shoots, roots, hairy roots) was based on published protocols [71, 72] with a slight modification. Briefly, 10 mL of n-hexane containing 5 % v/v ethyl acetate was used to extract 1 g of biomass in a cold (0 °C) sonication bath (PUL 125, Kerry Ultrasonics, UK) operated at 50 Hz for 30 min. The solvent from the extract was removed in vacuo and the residue re-suspended in 2 mL acetonitrile, which was then filtered through a 0.2 µm syringe filter (Fisher, UK) to remove waxes and other un-dissolved components. An aliquot of the filtrate placed in an HPLC vial was dissolved in the mobile phase and internal standard added for LC–MS/MS analysis.

#### Liquid chromatography method

The analysis was performed on an Aquity liquid chromatography unit coupled to a tandem quadruple detector (TQD) (Waters Corp., Milford, MA, USA). The liquid chromatography setup consisted of a binary pump, a cooling auto-sampler set at 10 °C with an injection loop of 10 µL. The column temperature was set at 30 °C and a Genesis^®^ Lightn C18 column (100 × 2.1 mm; 4 µm) (Grace, IL, USA) was used for the separation of the metabolites. The method by [73] was employed which consisting of a binary mobile phase of A: 0.1 % formic acid in water and B: 0.1 % formic acid in acetonitrile. Chromatographic separation was achieved on a linear gradient run of 0–7.0 min, 25–98 % B; 7–9.5 min, 98 % B; 9.5–10 min, 98–25 % B; 10–15 min, 25 % B; and a flow rate of 0.4 mL min^−1^.

#### Multiple reaction monitoring (MRM) method

The mass spectrometer was operated in MRM mode with positive electrospray ionization (ESI+) using an earlier method [73]. Briefly, the cone and de-solvation gas flow rates were set at 45 L h^−1^ and 800 L h^−1^, respectively, while the capillary voltage, the source and de-solvation temperatures were similar for all analytes at 28 kV, 150 and 350 °C, respectively. Cone and collision voltages were set at 24 and 7 V, respectively. A MRM transition of 283 → 219 + 229 + 247 + 265 was used for identification and quantification of artemisinin. The dwell time was automatically set at 0.161 s. Data were acquired by MassLynx V4.1 software and processed for quantification with QuanLynx V4.1 (Waters Corp., Milford, MA, USA).

### Analysis of metabolic pathway

Metabolic pathway of artemisinin production in *Artemisia annua* and *Artemisia dubia* and the effect of *rol* genes through which these genes enhance the production of artemisinin were analysed using different genes involved in artemisinin production, such as *CYP71AV1* (Cytochrome P450), *ADS* (Amorpha-4, 11-diene, synthase), *ALDH1* (Aldehyde dehydrogenase 1) and *TFAR1* (Trichome-Specific fatty acyl-CoA reductase 1) involved in trichome development and sesquiterpenoid biosynthesis.

### Quantitative real time PCR (qRT-PCR)

RNA extraction was performed using RNAqueous Small Scale Phenol-Free Total RNA Isolation Kit (Ambion) for small scale RNA isolation according to the manufacturer’s instructions. Frozen plant tissue powder (70 mg) was used for each RNA extraction. RNA (1 µg) was reverse transcribed using ThermoScript RT-PCR system (Invitrogen) primed with oligo (dT) _20_primer. The RNA was removed from the first strand cDNA by RNase treatment using RNase H (Invitrogen) according to the manufacturer’s instructions.

The qRT-PCR was performed by using following specific primers (Table 1). Primers were designed for the five genes using PrimerSelect (Lasergene; DNAStar) based on the cDNA sequences specific for the *Artemisia annua* available at the National Centre for Biotechnology Information. Primer pairs were designed with almost similar melting temperatures, to amplify 200–300-bp fragments, and checked for amplification and specificity by gel electrophoresis of PCR products.

**Table 1:** Primers used for genes (*ADS*, *CYP71AV1*, *ALDH1*, *TFAR1* and *ACTIN*) in qRT-PCR for artemisinin biosynthesis pathway analysis

No	Name	Forward primer	Reverse primer	Accession numbers
1	*TFAR1*	CCTTGGAGATCCTGAAGCTG	CGTTGGATTGTGCTGAACTG	GU733320
2	*ADS*	GGGAGATCAGTTTCTCATCTATGAA	CTTTTAGTAGTTGCCGCACTTCTT	JQ319661
3	*ALDH1*	CAGGAGCTAATGGAAGTTCTAAGTCAG	TTTCTTCCTTCGGCCACTGTTG	FJ809784
4	*CYP71AV1*	AGGGTAGGCATTCGCCGTCC	TCGAGTGGCCCTAACAACCTGC	HQ315834
5	*ACTIN*	ATCAGCAATACCAGGGAACATAGT	AGGTGCCCTGAGGTCTTGTTCC	EU531837

The real-time RT-PCR analysis was performed in an LightCycler^®^ 480 Real-Time PCR System (Roche Applied Sciences, Foster City, CA, USA), using the cDNAs as templates and the random primers for the analysed gene, as listed in Table 1. The actin gene was used as a reference gene. SYBR Green (SYBR Premix Ex Taq; TaKaRa) was used in the polymerase chain reaction (PCR) to quantify the amount of dsDNA. PCR was performed using 3 µl of the cDNA in a total of 20 µl reaction volume. The PCR conditions were 2 min at 95 °C, 30 s at 95 °C, 30 s at 60–62 °C, 1 min at 72 °C for 45 cycles, followed by 5 min at 72 °C. These conditions were chosen because none of the samples analysed reached at exponential phase at the end of the amplification. Expression analysis of each gene was confirmed in at least three independent RT-reactions using forward and reverse primers.

### Analysis of Trichome density

The number of glandular trichomes was determined at the adaxial side of three random pieces of fresh leaf material from each sample with an accurately determined leaf area of approximately 5 mm^2^ per leaf. Glandular trichomes were counted by using confocal microscope (Leica TCS SP5; Broadband Confocal, Leica Microsystems).

Each leaf was placed on a glass slide with three drops of Type F immersion liquid (Leica Microsystems CMs GmbH) with silicon vacuum grease at each corner below the cover slip to level the leaf. Clear nail polish sealed the cover slip to secure the sample and prevent evaporation. Using the confocal microscope three pictures of each leaf were taken and trichome density was analysed.

### Statistical analysis

Experiments were independently replicated at least three times and significant differences in artemisinin content, expression of artemisinin synthesis pathway genes and trichome density were evaluated using Welch’s two-sample *t* test (Welch, 1947).

## Results

**Fig. 2:**
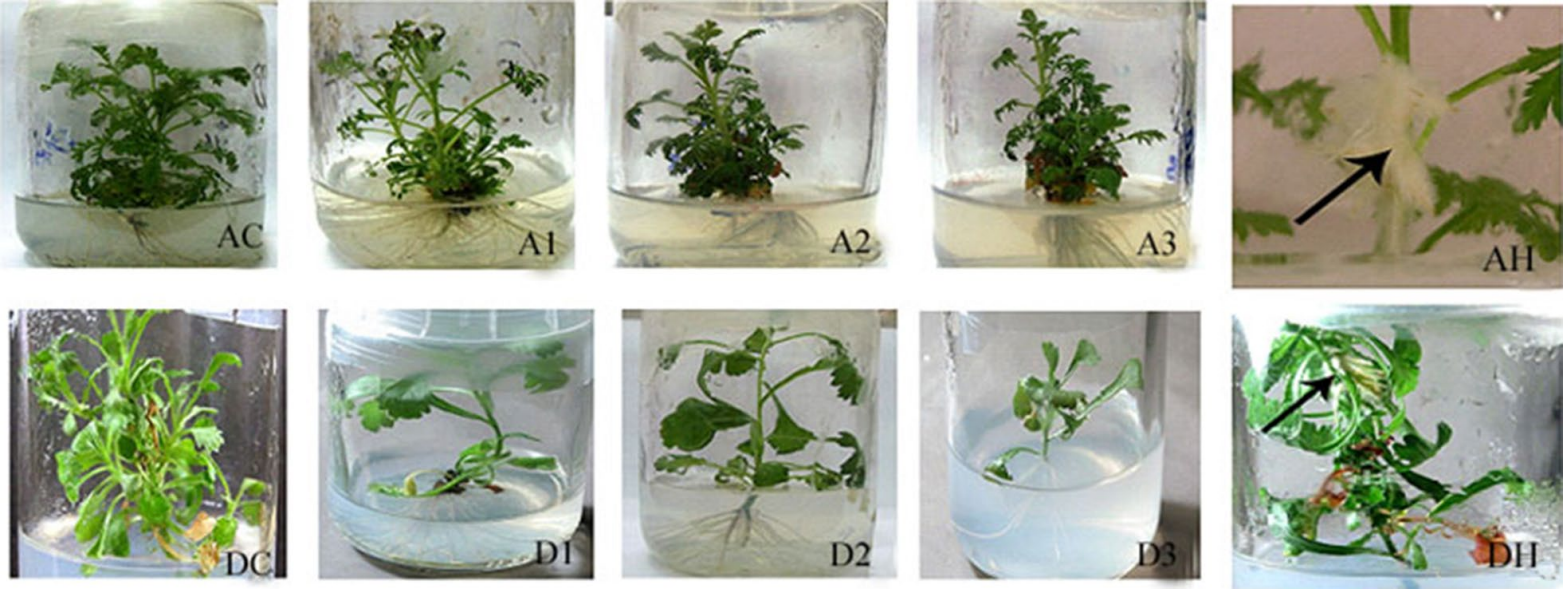
Comparison of transformed and untransformed plant transformed by *Artemisia tumefaciens.* *A1*–*A3* represents the transformed plants of *Artemisia annua;* *D1*–*D3* represents the transformed plants of *Artemisia dubia*. *AC* and *DC* are untransformed control plants of *Artemisia annua* and *Artemisia dubia,* respectively. *AH* represents the production of hairy roots from the plants of *Artemisia annua;* *DH* represents the production of hairy roots from the plants of *Artemisia dubia*

Three independent transgenic lines of *Artemisia annua* (A1, A2, and A3) and *Artemisia dubia* (D1, D2 and D3) were obtained by transformation with *Agrobacterium tumefacienes* and two independent transgenic lines of hairy roots were obtained by transformation with *Agrobacterium rhizogenes* (AH1 and AH2 of *Artemisia annua* and DH1 and DH2 of *Artemisia dubia*) carrying *rol ABC* genes (Fig. 2).

### Molecular analysis

**Fig. 3:**
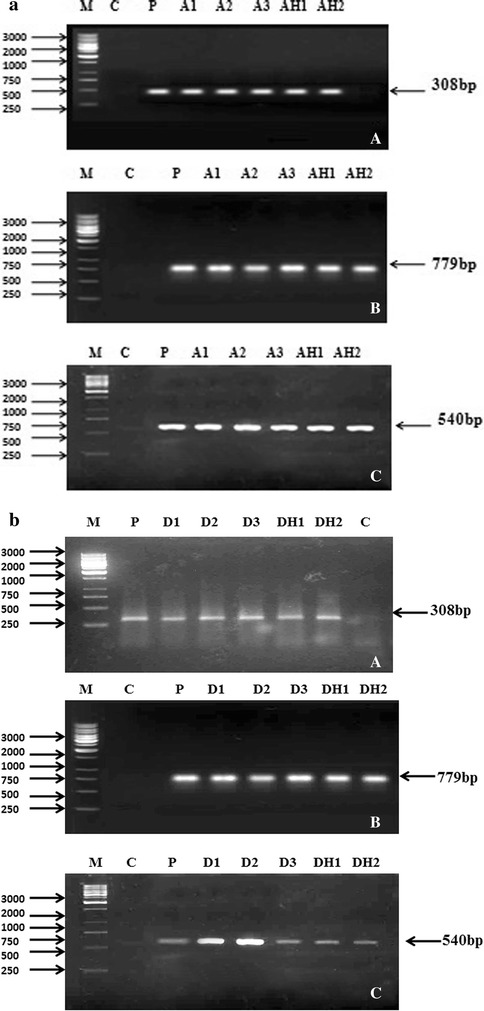
PCR analysis showing amplified products (**a**) *A1*–*A3* represents the plants transformed by *Agrobacterium tumefaciens* and *AH1*–*AH2* represents the plants transformed by *Agrobacterium rhizogenes* in *Artemisia annua* (**b**) *D1*–*D3* represents the plants transformed by *Agrobacterium tumefaciens* and *DH1*–*DH2* represents the plants transformed by *Agrobacterium rhizogenes* in *Artemisia dubia*. *Lane P* represents the plasmid DNA. *Lane C* refers to the non transformed control plants. *Lane M* corresponds to 1 kbp Ladder (Fermentas)

**Fig. 4:**
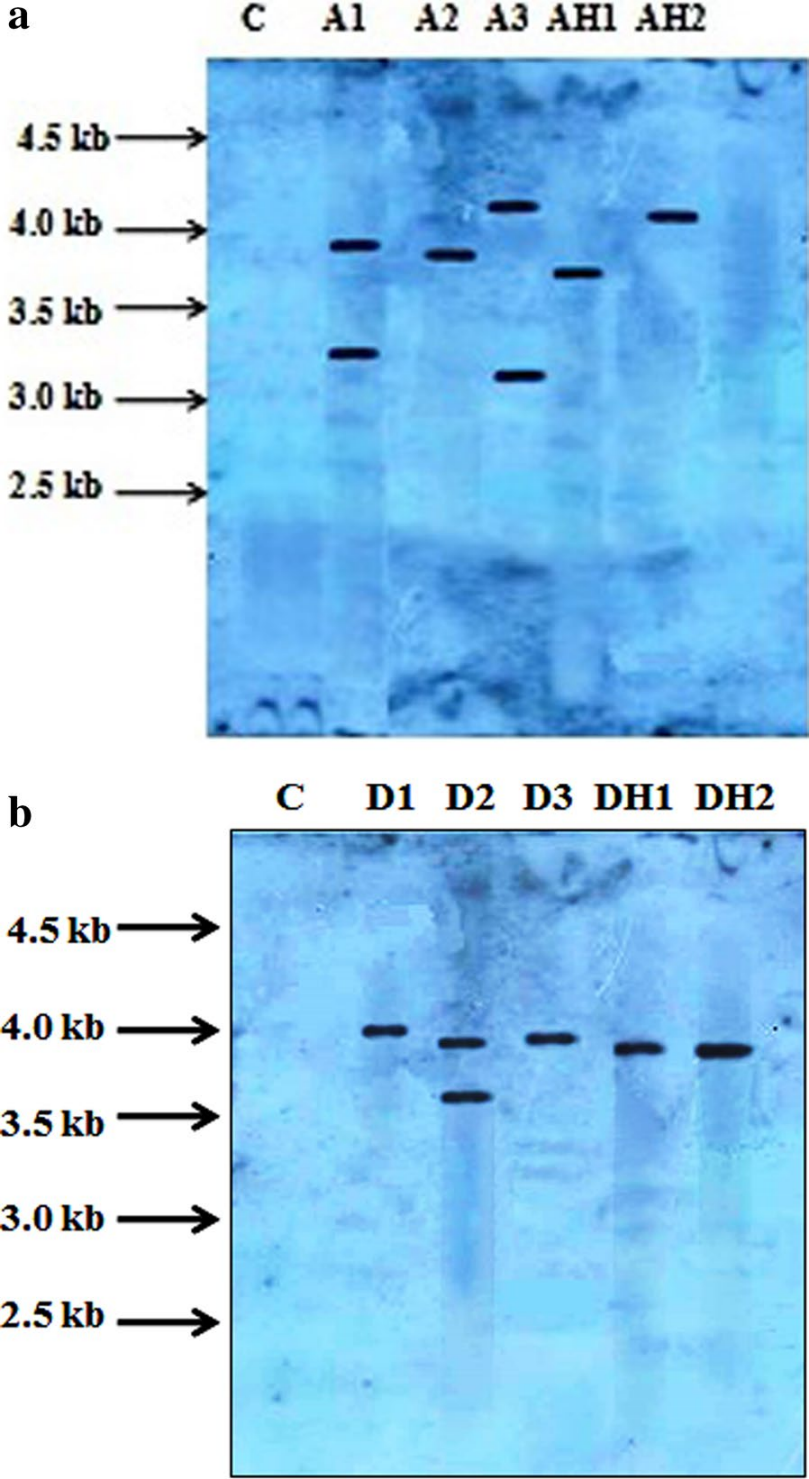
Southern blot analysis of (**a**) *Artemisia annua* (**b**) *Artemisia dubia*. C untransformed control plant, lanes 1–5 *rol ABC* transformed plants (*Artemisia annua*),* lanes 1*–*5* *rol ABC* transformed plants (*Artemisia dubia*),* A1*–*A3* represents the plants transformed by *Agrobacterium tumefaciens* and AH1-AH2 represents the plants transformed by *Agrobacterium rhizogenes* (*Artemisia annua*), D1-D3 represents the plants transformed by *Agrobacterium tumefaciens* and* DH1*–*DH2* represents the plants transformed by *Agrobacterium rhizogenes* (*Artemisia dubia*)

Molecular analysis of transformed plants/hairy roots was done with the help of PCR for *rol A, B* and *C* genes and the amplified products (308 bp, 779 bp, 540 bp respectively) were observed to confirm transformation. Same size amplified product was also obtained from the plasmid DNA of *Agrobacterium* strains. *Rol A, B* and *C* genes were detected in all transformed lines of shoots (A1, A2, A3, D1, D2, D3) roots (AR1, AR2, AR3, DR1, DR2, DR3) and hairy roots (AH1, AH2, DH1, DH2) as well as in plasmid DNA but not in control roots (Fig. 3a, b). Hybridization bands were detected in Southern blots with the *rol A* probe in the transgenic plants and hairy roots. In all the transformants, the inserted copy number was one except transgenic lines A1 and A3 of *Artemisia annua* and D2 of *Artemisia dubia* in which two copies of the inserted gene were observed (Fig. 4a, b). The results confirmed the integration of the *Agrobacterium* T-DNA in the genome of *Artemisia annua* and *Artemisia dubia* transgenic lines.

### Morphological characters

Considerable variation in the morphology of transgenic lines was observed as compared to controlled plants, like decrease in plant height with small and narrow leaves, terminal inflorescence and hard texture. The detailed morphological differences studied are listed in Table 2.

**Table 2:** Morphological differences observed among transgenic and control plants of *Artemisia annua* and *Artemisia dubia*

Morphological characters	Control plants	Transgenic plants
Plant height	87 cm ± 0.5	70 cm ± 0.3
Stem	Straight, unbranched and soft in texture	Branched and hard in texture
Leaves	Large size and broad	Small size and narrow
Inflorescence	Axial, without hairs	Terminal, excessive hairy

### Analysis of artemisinin content

**Fig. 5:**
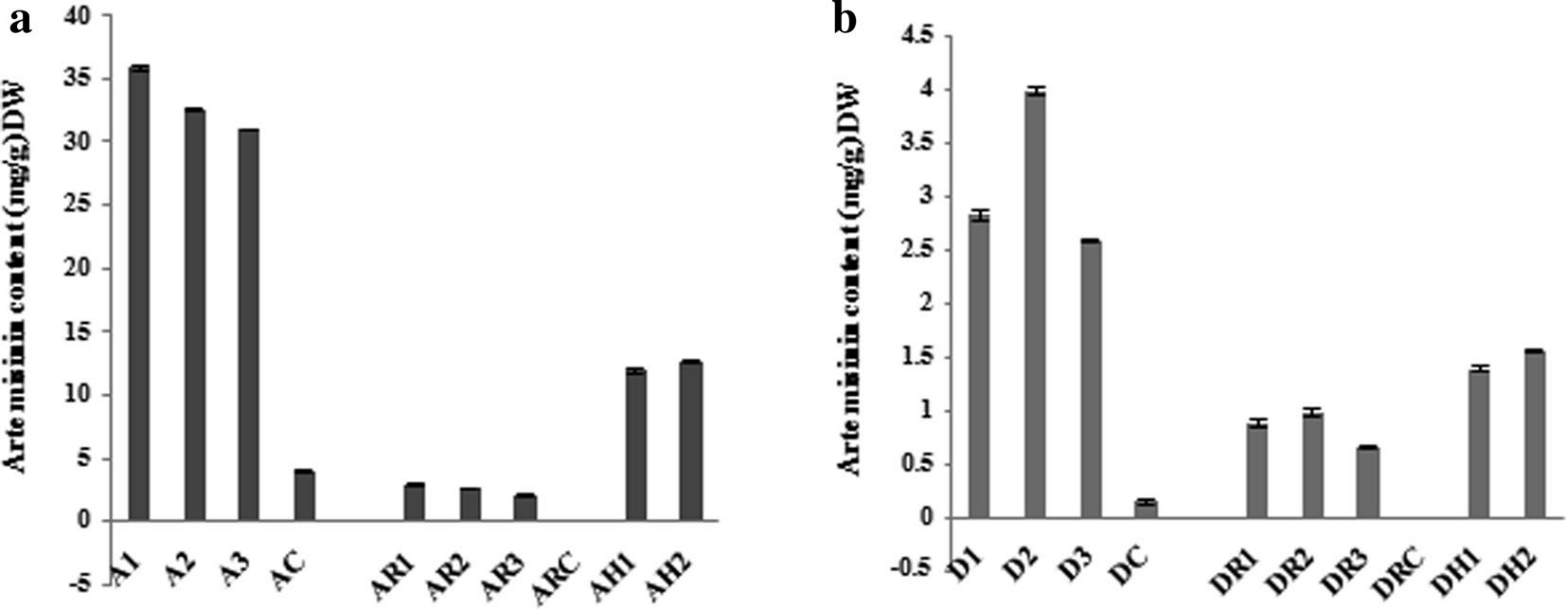
Artemisinin content in shoots, roots and hairy roots of transformed and un-transformed plants of *Artemisia dubia* and *Artemisia annua*. (**a**) Artemisinin content in shoots roots and hairy roots of *Artemisia annua.* *A1*,* A2* and* A3* represents leaf samples from three different transgenic lines,* AR1*,* AR2* and* AR3* represents roots of transgenic lines* A1*,* A2* and* A3* respectively,* AH1* and* AH2* represents two transgenic lines of hairy roots,* AC* and* ARC* represent control shoots and roots. (**b**) Artemisinin content in shoots, roots and hairy roots of *Artemisia dubia.* *D1*,* D2* and* D3* represent leaves from three transgenic lines.* DR1*,* DR2* and* DR3* represents transformed roots of transgenic lines* D1*,* D2* and* D3* respectively,* DH1* and* DH2* represents two transgenic lines of hairy roots,* DC* and* DRC* represent control shoots and roots. Both technical and biological replicates were performed. The average of three plants is shown together with error bars showing SD. Data was analyded for significant difference compared to the controls and all showed P < 0.001 with the control by Welch’s two samples t test

The average artemisinin content from three transgenic lines of *Artemisia annua* varied between tissues, the difference between the lines is shown (Fig. 5). In the shoots this was 33.12 mg/g dry weight (DW) compared to 3.94 mg/g DW in the controls shoots. Roots of transformed plants had an average artemisinin content of 2.72 mg/g DW while the amount found in transformed hairy roots was significantly greater (12.45 mg/g DW) compared to the control roots in which negligible amounts could be detected (0.02 mg/g DW). The average artemisinin in transgenic lines of *Artemisia dubia* was lower than *Artemisia annua.* Transgenic shoots of *Artemisia dubia* contained 2.32 mg/g DW compared to control shoots (0.11 mg/g DW), transformed plant roots (0.91 mg/g DW) and hairy roots (1.48 mg/g DW), whereas no artemisinin was found in control roots of *Artemisia dubia.*

### Analysis of metabolic pathway and trichome development

**Fig. 6:**
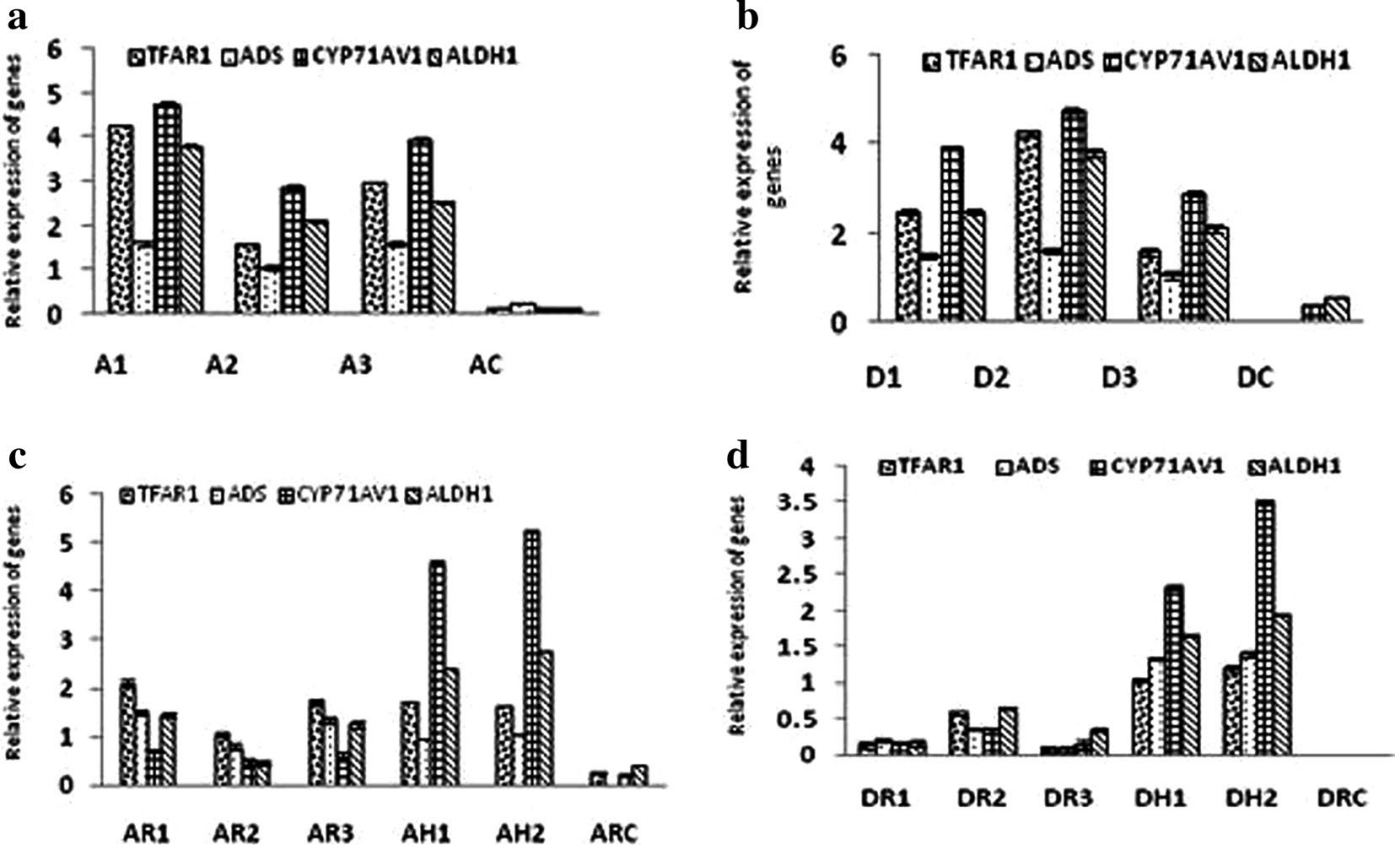
Expression of key genes *CYP71AV1* (Cytochrome P450), *ADS* (Amorpha-4, 11-diene, synthase), *ALDH1* (Aldehyde dehydrogenase 1) involved in artemisinin synthesis pathway of *Artemisia annua* and *Artemisia dubia* for artemisinin biosynthesis, and *TFAR1* (Trichome-Specific fatty acyl-CoA reductase 1) involved in trichome development and sesquiterpenoid biosynthesis. **a**. Expression of genes in leaves of *Artemisia annua.* *A1*,* A2* and* A3* represents three different transgenic lines of leaves,* AC* represents control shoots. **b**. Expression of genes in leaves of *Artemisia dubia.* *D1*,* D2* and* D3* represents three different transgenic lines of leaves, DC represents control shoots. **c**. Expression of genes in hairy roots, roots of transformed shoots of *Artemisia annua.* *AH1* and* AH2* represent two transgenic lines of hairy roots;* AR1*,* AR2* and* AR3* represent transformed roots of transgenic lines* A1*,* A2* and* A3* respectively.* ARC* represents control roots. **d**. Expression of genes in hairy roots, roots of transformed shoots of *Artemisia dubia.* *DH1* and *DH2* represent two transgenic lines of hairy roots;* DR1*,* DR2* and *DR3* represent transformed roots of transgenic lines *D1*,* D2* and* D3* respectively.* DRC* represents control roots. The actin gene was used as a house-keeping control. Both technical and biological replicates were performed. The average of three plants is shown together with error bars showing SD. Data analysed for significant difference at P < 0.001 with the control by Welch’s two samples t-test

The genes *ADS*, *CYP71AV1* and *ALDH1* are involved in the metabolic synthesis pathway of artemisinin and are involved in the conversion of FDP to dihydroartemisinic acid, which is a late precursor of artemisinin (Fig. 1). These key genes were used to determine how transformation with the *Agrobacterium rol ABC* genes affected the pathway in the different tissues of *Artemisia annua* and *Artemisia dubia.* Trichome-Specific fatty acyl-CoA reductase 1 (*TAFR1*) has been suggested to be involved in trichome development and sesquiterpenoid biosynthesis, both of which are important for artemisinin production. The expression of this gene was monitored to determine if trichome density might also be affected. Figure 6 shows expression levels of these genes in different tissues of both transformed and untransformed *Artemisia annua* and *Artemisia dubia*.

### Relative expression of genes involved in metabolic pathway of artemisinin biosynthesis

Pronounced changes in the expression of the artemisinin biosynthetic pathway genes were observed in all transgenic lines of *Artemisia annua* and *Artemisia dubia* transformed with *rol ABC* genes (Fig. 6a–d). The qPCR data presented here clearly demonstrate that the expression levels of *ADS*, *CYP71AV1* and *ALDH1* were significantly increased (P < 0.0001) in transformed plants of both *Artemisia* sp. compared to untransformed plants. Expression of artemisinin biosynthetic pathway genes was observed in hairy roots and roots of transformed plants compared to control roots of both *Artemisia annua* and *Artemisia dubia* in which low or negligible levels of expression are found. Expression levels of all the artemisinin biosynthetic genes were also significantly higher in transformed shoots compared to control plants. Interestingly, transformed *Artemisia dubia* showed levels, which were greater than, control *Artemisia annua* plants despite similar artemisinin content. Not all the genes examined exhibited the same increase in expression. *Cyp71AV1* appeared to show the greatest changes in relative expression in both shoots and hairy roots of both *Artemisia annua* and *Artemisia dubia* compared to the other genes. Relative expression of *CYP71AV1*gene was higher than that of the other genes. Its expression was significantly increased in transformed plants of both *Artemisia* species compared to untransformed plants, i.e. ~5 and ~120 times more in transformed leaves of *Artemisia annua* and *Artemisia dubia,* respectively. Roots of transformed plants in both *Artemisia* species showed significantly higher expression of this gene compared to control roots. The greatest difference in expression of this gene was found in hairy roots from both species even compared to transformed roots and control roots.

Relative expression of the ADS gene was generally lower than that of the other genes however the difference in its expression compared to the control was the highest. i.e. ~11 and ~270 greater in transformed leaves of *Artemisia annua* and *Artemisia Dubia,* respectively. Roots from these plants showed even larger differences in the expression of this gene compared to the controls. In *Artemisia annua,* the levels of expression of ADS was comparable between transformed roots and hairy roots, but in *Artemisia dubia* there was a significant difference in the expression of this gene.

The ALDH1 gene showed high expression in transformed plants of both *Artemisia* species when compared to untransformed plants, i.e. ~7 and ~5 times more in transformed leaves of *Artemisia annua* and *Artemisia dubia,* respectively. Roots of these transformed plants also showed greater differences in expression of this gene in *Artemisia annua* compared to control, in contrast in *Artemisia dubia* the level of expression of this gene was comparatively low. However expression of this gene showed significant differences in hairy roots of both *Artemisia* species compared to control roots.

### Trichome development

A significant increase in expression of TFAR1 was detected in transformed plants of both *Artemisia* species compared to untransformed plants, i.e. ~10 and ~300 times more in transformed leaves of *Artemisia annua* and *Artemisia dubia,* respectively. Unexpectedly this gene was expressed in abundance in transformed roots and hairy roots of both *Artemisia* species. The levels of expression were greater than that seen in control shoots. Although this gene has been suggested to be responsible for trichome density it showed significant differences in expression even in transformed roots and hairy roots compared to control roots.

### Analysis of trichome density

**Fig. 7:**
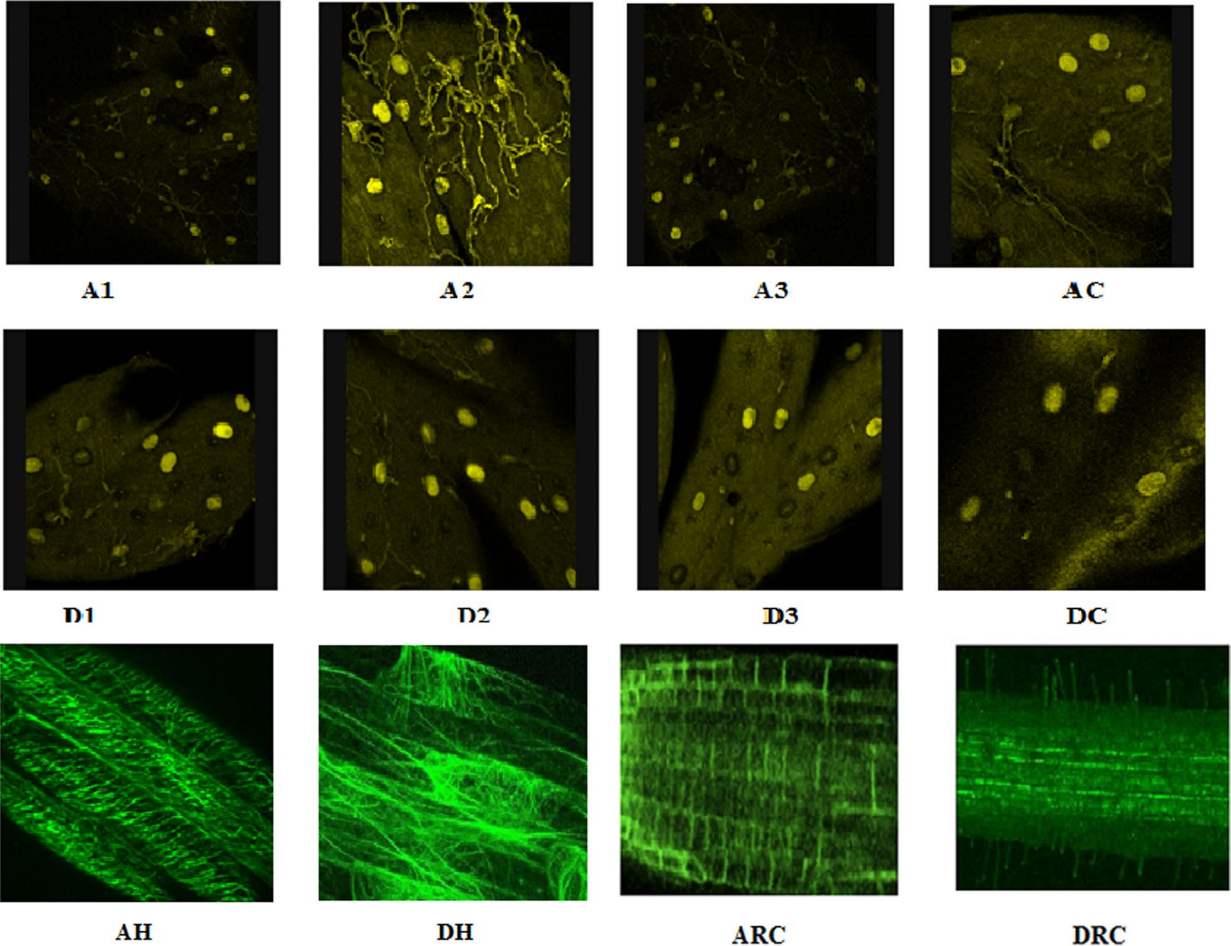
Comparison of trichome density in different tissues of transformed and untransformed plants of *Artemisia annua* and *Artemisia dubia.* Trichome density was analysed by using confocal microscope.* A1*,* A2* and* A3* represents three transgenic lines of leaves in *Artemisia annua*, AH represents hairy roots; AC and ARC represent control shoots and roots of *Artemisia annua,* respectively.* D1*,* D2* and* D3* represent three transgenic lines of leaves in *Artemisia dubia*,* DH* represents hairy roots,* DC* and* DRC* represent control shoots and roots of *Artemisia dubia,* respectively. Both technical and biological replicates were performed

**Fig. 8:**
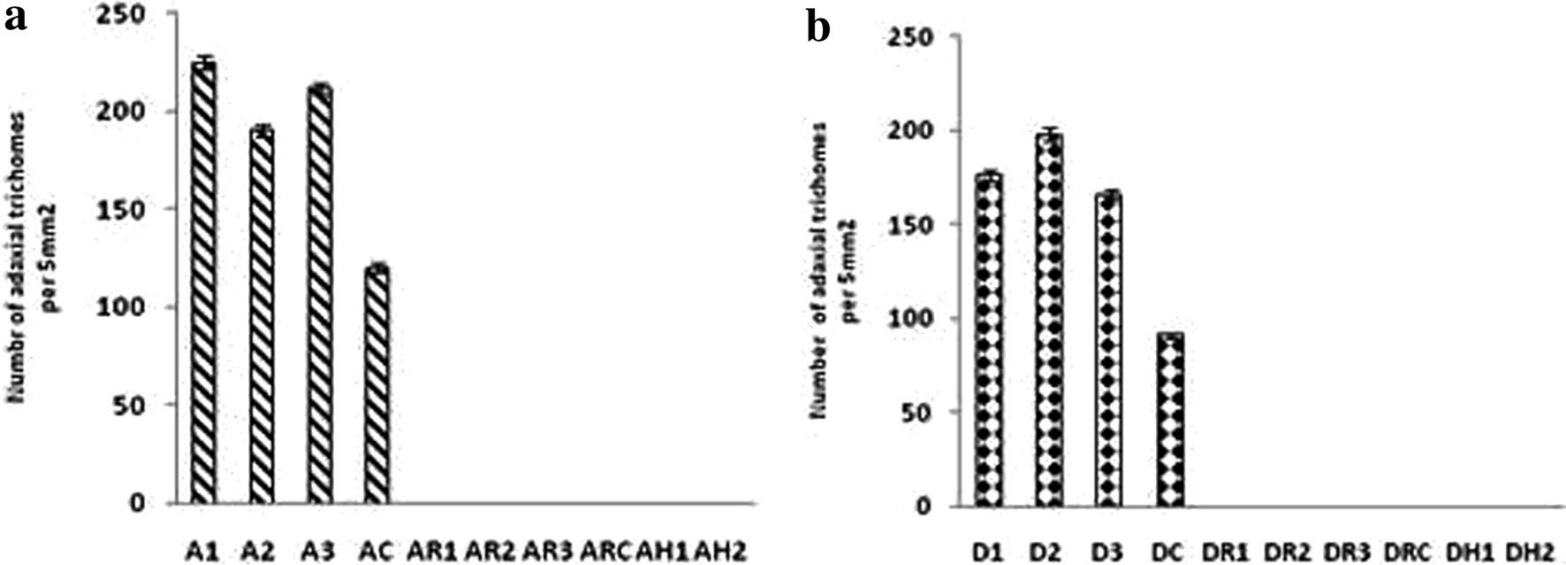
Comparative analysis of trichome density in transformed and untransformed plants of *Artemisia annua* and *Artemisia dubia.* **a** Trichome density in shoots roots and hairy roots of *Artemisia annua.* *A1*,* A2* and* A3* represents three different transgenic lines of leaves,* AR1*,* AR2* and *AR3* represents transformed roots of transgenic lines* A1*,* A2*,* A3* respectively,* AH1* and* AH2* represents two transgenic lines of hairy roots, AC and ARC represent control shoots and roots of respectively. **b**. Trichome density in shoots roots and hairy roots of *Artemisia dubia.* D1, D2 and D3 represents three transgenic lines of leaves, DR1, DR2 and DR3 represents transformed roots of transgenic lines D1, D2, D3, respectively, DH1 and DH2 represents two transgenic lines of hairy roots, DC and DRC represent control shoots and roots of respectively. Both technical and biological replicates were performed. The average of three plants is shown together with *error bars* showing SD. Data analysed for significant difference at P < 0.001 with the control by Welch’s two samples t test

Trichome density was assessed using ESEM at low resolution and is shown in Fig. 7. Transformed leaves of *Artemisia annua* plants produced more trichomes (~222 trichomes/5 mm^2^) as compared to the control leaves (~120 trichomes/5 mm^2^). Transformed leaves of *Artemisia dubia* also produced more trichomes (~173 trichomes/5mm^2^) compared to control leaves (~173 trichomes/5mm^2^) In contrast, the hairy roots, transformed and untransformed roots from both *Artemisia* species showed no trichome production (Figs. 7, 8).

Transformation also increased hairiness in transformed plants along with increase in trichome density compared to non-transformed plants. Transformed plants produced denser network of hair on its surface compared to non-transformed plants (Fig. 7).

## Discussion

Artemisinin analysis was carried out on dried plant material (shoots, roots and hairy roots) of transformed and non-transformed plants of *Artemisia annua* and *Artemisia dubia*. The tissue assayed was similar to that from *Artemisia* species in previous studies [5, 41, 74–77]. Control plants in the current study contained similar amounts of artemisinin when compared to these studies on the vegetative stage. In the present study, 0.39 % DW artemisinin in control plants of *Artemisia annua* and 0.01 % DW artemisinin in *Artemisia dubia*, while Wallaart et al. [11] found 0.1–0.5 % DW artemisinin in control plants of *Artemisia annua*, Weathers et al. [78] found 0.2–0.5 % artemisinin in vegetative leaves and Mannan et al. [72] found 0.44 % DW artemisinin in *Artemisia annua* leaves and 0.05 % DW in leaves of *Artemisia dubia.*

Transformed shoots, roots of transformed shoots and hairy roots of *Artemisia annua* and *Artemisia dubia* all contained significantly more artemisinin when compared to the controls. The highest concentration of artemisinin were observed in transgenic leaves of both *Artemisia* species (i.e. ~9 and ~25 fold increase in leaves of *Artemisia annua* and *Artemisia dubia,* respectively, compared to controls). Other researchers have previously reported that transformation of different *Artemisia* species results in an increase in production of artemisinin within the plant. Vergauwe et al. [79] reported that *Agrobacterium tumefacienes* transformation with EHA101 (pTJK136) strain and C58C1RifR (pGV2260) (pGSJ780BAT) strain of *Artemisia annua* resulted in an increase in artemisinin content (0.42 %) and its metabolites. He et al. [63] also reported 0.36 % increase in artemisinin amount in transformed leaves of *Artemisia annua* as compared to non-transformed plants. Similarly, Adbin et al. [47] reported the presence of artemisinin, ranging from 0.01 to 0.80 %, in leaves and flowers of transformed *Artemisia annua* and by overexpression of genes coding for enzymes associated with the rate limiting steps of artemisinin biosynthesis pathway. Han et al. [80] reported that artemisinin production increased by up regulation of FDS through genetic transformation of *Artemisia annua* with different genes. Overexpression of *Gossypium arboretum* FPS in hairy roots and transgenic plants resulted in three- to four-fold, and two- to three-folds increased artemisinin content, respectively [81, 82]. The results achieved in this study are significantly higher, with increase of 12-fold or greater being detected depending on the tissue examined.

A meaningful increase in artemisinin content was observed in hairy roots and roots of transformed shoots of *Artemisia annua* and *Artemisia dubia* as compared to control, similarly roots of transformed plants in both *Artemisia* species also showed significant increase in artemisinin content compared to control roots. An earlier report shows that *Agrobacterium rhizogenes* mediated transformation increased the level of secondary metabolites in plants [83]. Artemisinin production (0.42 %) has been reported in hairy roots culture of *Artemisia annua* [62]. Mannan et al. [7] reported the production of artemisinin by hairy roots in *Artemisia dubia* (0.603 %) and *Artemisia indica* (0.0026 %) with *Agrobacterium rhizogenes* strain LBA9402. A significant increase in artemisinin content in hairy roots culture of *Artemisia Annua* was also reported [84]. The concentration of artemisinin in roots of untransformed *Artemisia annua* and *Artemisia dubia* is almost negligible (Fig. 5a, b). These findings agree with others [50, 85] who found very low artemisinin concentrations (0.003 %) in root of *Artemisia annua.* Other studies did not detect artemisinin within roots of *Artemisia annua* perhaps due to the sensitivity of the analysis carried out [49, 52, 55, 63, 86]. The current study also found low (*Artemisia annua*) or zero (*Artemisia dubia*) artemisinin concentration in untransformed roots. Transformed plants, hairy roots and roots of transformed shoots of *Artemisia dubia* and *Artemisia annua* were all found to contain significantly higher amounts of artemisinin.

The results obtained clearly show that the roots of transformed plants and hairy roots both contain around 1.5 % artemisinin. This is only slightly less than that found in leaves of *Artemisia annua* and significantly higher than that found in leaf material from *Artemisia dubia*. It has been reported that exogenous artemisinin is capable of inhibiting root growth at concentrations greater than 10 μg/ml^−1^ [87]. The study showed that hairy roots were capable of producing more than 15 μg g^−1^ DW. This would suggest that there are certain cells within the roots which are acting as storage compartments for the artemisinin produced. Mannan et al. [12] had shown that roots of *Artemisia* plants are capable of producing artemisinin. Interestingly the roots do not contain trichomes; hair cells are however present and it is possible that these cells are also involved in the production and storage of artemisinin. The number of hair cells also appeared to be present in greater abundance on the leaves of transformed plants (Fig. 7).

Presented study found that expression of all the genes involved in artemisinin synthesis pathway significantly increased in all transgenic lines of *Artemisia dubia* and *Artemisia annua* transformed with *rol ABC* genes as compared to non-transformed plants (Fig. 6a, b, c, d), which is in clear agreement with the data obtained for artemisinin content.

Expression of ADS and CYP71AV1, of which the corresponding proteins catalyze the formation of amorpha-4, 11-diene and its ultimate conversion to dihydroartemisinic acid, was increased significantly in transformed shoots of *Artemisia annua* and *Artemisia dubia,* respectively. Similarly significant differences in expression of these genes were observed in hairy roots and roots of transformed plants of both *Artemisia annua* and *Artemisia dubia* compared to untransformed roots. The expression of CYP71AVI corresponds to that found by [88] however the result for the ADS gene differs as they were unable to show ADS gene expression in hairy roots. The ALDH1 gene which catalyses the oxidation of artemisinic and dihydroartemisinic aldehydes was also highly expressed in transformed plants of both *Artemisia* species as compared to untransformed plants, i.e. ~7 and ~5 times more in transformed leaves of *Artemisia annua* and *Artemisia dubia*, respectively, ~5times in roots of transformed shoots of *Artemisia annua* and in *Artemisia dubia* this increased from negligible amounts to significant levels, with similar increases in hairy roots compared to untransformed roots (Fig. 6a–d). Although all these genes showed increased expression in transformed plants and hairy roots, however induction of CYP71AV1 and ALDH1 was much more compared to the ADS gene.

TFAR1, known to stimulate trichome development and to catalyze sesquiterpenoid biosynthesis [17] showed significantly increased expression in all transgenic lines of *Artemisia annua* and *Artemisia dubia* (Fig. 6). Density of trichomes were also measured in transformed and non-transformed plants and found that transformed leaves of *Artemisia annua* and *Artemisia dubia* have a higher density of trichomes compared to leaves of non-transformed plants. Despite close examination no trichomes were found on roots and hairy roots cultures of either *Artemisia* species but they were shown to contain significant amount of artemisinin as shown in Fig. 5a, b. Olofsson et al. [88] found that leaves and flower buds of *Artemisia annua* produce more trichomes than other parts of the plant and also found that roots do not have trichomes. Although the pathway for the synthesis of artemisinin is well known [89], it has been widely assumed that the trichomes are the only source of artemisinin production in the plant [40, 50, 57, 58] despite several papers describing its presence within roots [12, 51, 56, 60], reports that the artemisinin content of the plant is associated with a rise in trichome density, but also continued to rise after a collapse in the trichome populations. The results of present study found that the trichome density in *Artemisia annua* is increased by 10 % following transformation however the artemisinin content increased by approximately 10 times. In *Artemisia dubia,* trichome density was increased by 9 % following transformation and artemisinin content was increased by approximately 30 times (Figs. 5a, b, 7, 8). These results suggest a possible dissociation between trichome density and artemisinin content. It is unclear if the genes involved in artemisinin production are expressed elsewhere in the leaves [90]. It has been previously suggested that the plant was unable to support concentrations higher than this and that the trichomes capacity for storing artemisinin is limited [91]. The current study shows that the plants are capable of producing and storing much higher amounts of artemisinin than previously thought both in leaves and in roots. This study shows significant amount of artemisinin production in transformed roots of *Artemisia annua* and *Artemisia dubia,* and a small amount of artemisinin in untransformed roots of *Artemisia annua*. The detection of significantly raised levels of expression of the genes involved in artemisinin in transformed roots associated with the detection of significant amounts of artemisinin in these tissues suggest that synthesis is occurring elsewhere in the plant as suggested by Duke et al. [90]. It should now be possible to determine how roots produce artemisinin hence elucidating how production could be improved.

Baldi and Dixit [92] and Wang et al. [93] have reported that artemisinin production in plants and culture cells is increased after treatment with methyl Jasmonate; another report examined the effect of cytokinin, gibberelin and jasmonate on artemisinin production [17]. Similarly, Arsenault et al. [94] described the effect of sugars on the production of artemisinin in *Artemisia annua*. Current study deal with the transformation of *Artemisia annua* and *Artemisia dubia* plants with *rol ABC* genes of *Agrobacterium tumefaciens* and *Agrobacterium rhizogenes,* which are responsible for enhancing production of secondary metabolites in plants. The exact mechanism for the action of the *rol* genes is still far from clear [26], it has been suggested that they act through the stimulation of the plants defense response which includes induction of many of the hormonal pathways described above. Many secondary metabolites are thought to be have evolved to protect plants as they expanded to fill different evolutionary niches. It has been suggested that artemisinin is an example of such a metabolite as it acts to prevent predation of the plant by herbivores and it is stimulated by pruning prior to harvest [95]. This may explain why the transformation with *Agrobacterium rol* genes has such a significant effect on the amount of artemisinin produced by the transformed plants. Stimulation of the synthesis of artemisinin within these plants has also allowed us to alter the perception of how and where artemisinin is produced within the plants. These findings will help to develop better strategies to increase the production of this valuable therapeutic drug.

## Conclusion

Transformation of *Artemisia annua* and *Artemisia dubia* with *rol ABC* genes results in the enhancement of secondary metabolites particularly the artemisinin and its derivatives. Moreover, the level of transcripts of the *rolABC* genes gene found in transgenics also correlate with their artemisinin accumulation pattern. This report covers the impact these genes have had on the capability of the plants to synthesize the anti-malarial drug. Analysis of genes involved in the synthetic pathways and comparisons of trichome density between transformed and non-transformed plants have been carried out. Significantly, an increase in trichome numbers and increased expression of biosynthetic pathway genes correlated with increased artemisinin content. This data allow us to present a novel interpretation of artemisinin production and how stimulation of the pathways by the *rol ABC* genes can enhance artemisinin production stimulating both the biosynthetic pathways and trichome development in transformed plants of *Artemisia dubia* and *Artemisia annua*. The insight this study has made into how and where artemisinin is produced within the plants will provide vital information that is required to increase production of artemisinin to meet the increasing demand of drug manufacturers due to its multi pharmacological importance.

## Abbreviations

*CYP71AV1*: cytochrome P450
*ADS*: amorpha-4 11-64 diene synthase
*ALDH1*: aldehyde dehydrogenase
*TFAR1*: trichome-specific fatty acyl-CoA reductase 1
